# Efficiency of Malaysian states in managing the COVID-19 outbreak in 2020 and 2021

**DOI:** 10.1371/journal.pone.0275754

**Published:** 2022-10-26

**Authors:** Abdul Rahim Isnain, Nazri Che Dom, Samsuri Abdullah, Nopadol Precha, Hasber Salim

**Affiliations:** 1 Centre of Environmental Health & Safety studies, Faculty of Health Sciences, Universiti Teknologi MARA (UiTM), UITM Cawangan Selangor, Puncak Alam, Selangor, Malaysia; 2 Integrated Mosquito Research Group (I-MeRGe), Universiti Teknologi MARA (UiTM), UITM Cawangan Selangor, Puncak Alam, Selangor, Malaysia; 3 Institute for Biodiversity and Sustainable Development (IBSD), Universiti Teknologi MARA, Shah Alam, Selangor, Malaysia; 4 Faculty of Ocean Engineering Technology and Informatics, Universiti Malaysia Terengganu, Kuala Nerus, Terengganu, Malaysia; 5 Department of Environmental Health and Technology, School of Public Health, Walailak University, Nakhon Si Thammarat, Thailand; 6 School of Biological Sciences, Universiti Sains Malaysia, Minden Penang, Malaysia; University of Siena: Universita degli Studi di Siena, ITALY

## Abstract

**Introduction:**

Many developing countries have drastically imbalanced health systems in different regions. The COVID-19 outbreak posed a further challenge as hospital structures, equipped with doctors, critical care units and respirators, were not available to a sufficient extent in all regions.

**Objective:**

This study is a descriptive study on the efficiency of Malaysian states in facing the COVID-19 outbreak.

**Methodology:**

The efficiency of all Malaysian states was measured using Data Envelopment Analysis in which each state’s Score of COVID Index (SCI) was quantified. The SCI of these states were then further compared between the year 2020 and 2021. A greater disparity would indicate a decline in the performance of a state over time, where nearly all the states in Malaysia experienced an increase in the score of COVID Index (SCI).

**Result:**

This study found that the central region was the most affected, since all the three states in the region (Selangor, Federal Territory of Kuala Lumpur, and Federal Territory of Putrajaya) showed a situation of inadequacy (SCI: >0.75) due to the COVID-19 outbreak.

**Conclusion:**

The ranking of Malaysia’s states according to their vulnerability to an outbreak of COVID-19 is vitally significant for the purposes of assisting the government and policymakers in planning their responses to the outbreak and ensuring that resources are distributed appropriately.

## Introduction

COVID-19 is expected to cause as much as or more human suffering than any other communicable disease on the planet. The study on the efficiency of COVID-19 management has been conducted previously [1–8]. These studies used the Data Envelopment Analysis to measure the efficiency of different Decision-Making Units (DMUs). These DMUs can either be countries, states or regions depending on the researcher’s selection. Each DMUs have its own inputs or outputs, which were also predefined by the researchers. Dlouhy [2] compared the efficiency of 38 DMUs (38 countries) by using 3 inputs (physicians, nurses and hospital beds) and 2 outputs (population and life expectancy). Breitenbach et al., [1] fixed their DMUs by selecting 36 countries which accounted for 90% of all infections and fatalities worldwide and established 3 inputs (number of tests, number of physicians and nurses and health expenditure) as well as 3 outputs (recovery rate, death rate and infection rate) in their study. Meanwhile, instead of comparing different countries as DMUs, Mariano et al., [6] compared the efficiency of federal units and states within one country (Brazil) by using 3 inputs (number of doctors, number of respirators and number of clinical beds) and 1 output (number of deaths). Based on their distinct inputs and outputs, efficiency analysis aids in comparing and ranking the efficiency level of these different DMUs.

The study of efficiency in handling the Covid-19 outbreak using the COVID Index was used by Ferraz et al [9]. They use COVID Index to determine whether hospital infrastructures in 543 Brazilian microregions are sufficient to handle COVID-19 and to determine whether appropriate public policies have been adopted. To quantify the efficiency of different states in controlling the COVID-19 pandemic, the study used the Data Envelopment Analysis (DEA) in which each state’s DEA scores were measured. In the study, the DEA score was termed as COVID Index.

In this study, a data envelopment analysis was used to build a COVID Index to test whether the healthcare structures in all Malaysian states were competent to cope with the COVID-19 outbreak. This index was introduced by Ferraz et al. [9]. The efficiency of each Malaysian state in managing the COVID-19 outbreak was further compared between the years 2020 and 2021. This analysis determines the ratio between the linear combination of outputs to the linear combination of inputs for every unit to calculate their relative efficiency. This study chose five healthcare resource variables as the inputs, namely, hospital beds, Intensive Care Unit (ICU) beds, COVID-19 Quarantine and Treatment Centre (CQTC) beds, number of physicians, and number of nurses. Meanwhile, the outputs chosen were the number of COVID-19 cases and COVID-19 mortality.

## Methods

### Study population

The research sites were spread out across the all states of Malaysia. The comparative efficiency of the Malaysian states was determined using primary data from these all states. The states studied were divided into six regions: the Northern Region (which included Perlis, Kedah, Penang, and Perak), the East Coast Region (which included Kelantan, Terengganu, and Pahang), the Central Region (which included Selangor, Kuala Lumpur, and Putrajaya), the Western Region (which included Negeri Sembilan and Malacca), the Southern Region (Johor and Pahang), and lastly, the region of East Malaysia (Sabah, Sarawak and Labuan).

### Study design

This was a descriptive study on the vulnerability of the Malaysian states in facing the COVID-19 outbreak. This research was primarily derived from the work of [9]. The time period that was considered for this study was from 25 January 2020 until 31 December 2021. The healthcare resources during the epidemic were studied using five variables as the DEA inputs from private and public hospitals. The input parameters included the number of hospital beds, number of ICU beds, number of CQTC beds, number of physicians, and number of nurses. The outputs represented two variables: the number of COVID-19 cases and COVID-19 mortality. The data were obtained in a digital format from reputable sources that were easily accessible to the public. All the input and output parameters are listed in Table 1.

**Table 1:** Study variables.

Variable	Classification	Data collection description	Reference
Number of hospital beds	Input	The total number of non-ICU beds in hospitals and health centres in each state that were regularly maintained and available for patient care.	[13]
Number of ICU beds	Input	The total number of ICU beds in hospitals and health centres in each state that were regularly maintained and available for patient care	-
Number of CQTC beds	Input	The total number of COVID-19 Quarantine and Treatment Centre beds in each state that were regularly maintained and available for patient care.	-
Number of physicians	Input	The physician-to-population ratio of 1:1000 was used to calculate the total number of registered medical physicians in each state.	[14]
Number of nurses	Input	The total number of registered medical nurses and midwives in each state was calculated using a nurse-to-population ratio of 1:1000	[15]
Number of COVID-19 cases	Output	The total number of COVID-19 cases in each state divided by its total population and multiplied by 10,000.	-
Covid -19 mortality	Output	The total number of deaths due to COVID-19 in each state divided by its total population and multiplied by 10, 000.	-

### Data collection & management

Geometric information of Malaysia in the form of shapefile was acquired from a freely available source [10] through https://earthworks.stanford.edu/catalog/stanford-qg469kj1734. The data on the healthcare resources or inputs were obtained from open-access sources. The number of beds was obtained from the open-source data of the Malaysian MOH COVID-19. This open source at https://github.com/MoH-Malaysia/covid19-public also provided the data on COVID-19 cases and COVID-19 mortality, which were the outputs in this study. The information was gathered for the years 2020 and 2021. The data on beds from this source was in the form of a timeline, with daily information of the number of beds from 25 January 2020 until 31 December 2021. The information was provided as a csv file, which was then converted to Excel and sorted to display the number of beds by state and year. The pivot table function in the Excel software was utilized to produce this result. Meanwhile, the output data regarding the number of physicians and nurses in 2020 were obtained from health indicator reports released on the official portal of the Malaysian MOH [11]. However, at the time this research was published, the data for the year 2021 had yet to be released. Therefore, in order to fill this data gap, a linear forecast for the data in 2021 was generated using the data from 2016 through 2021 as predictors [12].

### Data analysis

A data envelopment analysis (DEA) was used to determine the efficiency of each Malaysian state in managing the COVID-19 outbreak. Originally, a higher DEA efficiency score would indicate greater efficiency. In this study, however, an inverted DEA was used, whereby scores closer to 1 would indicate a lower efficiency and scores closer to 0 would indicate greater efficiency [16]. This inverted DEA score was thereafter known as the COVID Index. Next, the states in Malaysia were ranked according to their COVID Index scores. This study continued by comparing the COVID Index scores for 2020 and 2021. A DEA is a linear programming-based mathematical technique created by Charnes et al. [16]. It is a non-parametric way of measuring the relative efficiency of a group of observed units with similar characteristics. These units are known as the decision-making units (DMUs). In this study, the DMUs were the different states in Malaysia (n = 16). As for the characteristics of each unit, they were known as the inputs and outputs. This analysis determines the ratio between the linear combination of outputs and the linear combination of inputs for every unit to calculate their relative efficiency. In other words, a DEA determines the maximum number of outputs per unit of inputs that can be created. The inverted DEA was employed to explain the relationship between healthcare resources and the COVID-19 outbreak. Therefore, the lower the performance, the higher the efficiency score. In other words, the regions with the weakest performance were rated the highest. The range for the DEA measurement of the efficiency of the DMUs is [0, 1]. If the efficiency value of a DMU is 1, then, it is efficient; otherwise, it is inefficient. The COVID Index is a relative metric that utilizes an inverted DEA. This means the closer to zero the value, the better was the state’s use of healthcare resources to combat the COVID-19 epidemic. The closer the value to one, the worse the state was at utilizing healthcare resources to combat COVID-19. As illustrated in Table 2, the COVID Index suggested four distinct performance scenarios. These categories were utilized by Ferraz et al. [9] and helps to explain the state’s level of efficiency. States with an adequate situation have the right number of resources to treat COVID-19-infected patients. These states might be able to help other states without affecting their own health care resources. For instance, a state categorized as adequate could accept patients from neighboring states or provide them with human resources and respirators. In satisfactory states, adequate resources exist to combat the coronavirus epidemic. States requiring attention must be monitored by the authorities in order to prevent additional coronavirus infections and deaths, and their healthcare capacity cannot be reduced. States that are categorized as inadequate have the worst conditions among the states evaluated. Consequently, they must obtain resources from new investments or relocated equipment, as well as human resources from states in an adequate situation.

**Table 2:** Covid index categories.

Score of Covid Index (SCI)	Categories
0.00 *≥*0.24	Adequate
0.25 *≥*0.50	Satisfactory
0.51 *≥*0.74	Attention
0.75 *≥1*.*00*	Inadequate

Note: The closer the value to 1, the worse the state’s performance in utilizing healthcare resources to combat COVID-19.

Using inputs, outputs and the geometrical data, Covid Index visualization was produced using the RStudio software [17]. COVID Index contributed by aggregately evaluating the usage of infrastructure and human capital to combat the COVID-19 outbreak.

## Results

**Table 3** presents the descriptive data for inputs and outputs used in this study. The data shows that 3 out of 5 inputs had high standard deviations ranging from 366 to 3417.57 during the years 2020 and 2021. The inputs with high standard deviations were the number of hospital beds, number of ICU beds and number of CQTC beds. The large standard deviations suggested that the number of hospital beds, number of ICU beds and number of CQTC beds across the states in Malaysia varied significantly from the mean. This was evident from the large disparity between the minimum and maximum number of hospital beds, number of ICU beds and number of CQTC beds. The average number of these three inputs also increased about 3-fold in 2021 compared to in 2020.

**Table 3:** Descriptive statistics of inputs and outputs for years 2020 and 2021.

	2020				2021			
Input & Output	Minimum	Maximum	Mean	Standard deviation	Minimum	Maximum	Mean	Standard deviation
**Input:**								
Number of Hospital Beds	0	1264	497	366	106	4713	1313.06	1154.32
Number of ICU Beds	0	133	43	39	11	380	122.25	95.23
Number of PKRC Beds	0	9985	1792	3111	0	11345	3392.81	3417.57
Physician to population ratio	1	13	3	3	1.43	14.97	3.37	3.36
Nurse to population ratio	2	14	4	3	2.16	12.18	4.16	2.75
**Outputs:**								
Number of Cases per 10,000 population	0	0.6	0.1	0.1	0.353	2.314	1.048	0.511
Number of Deaths due to COVID-19 per 10,000 population	0	0	0	0	0.016	0.151	0.086	0.041

**Fig 1:**
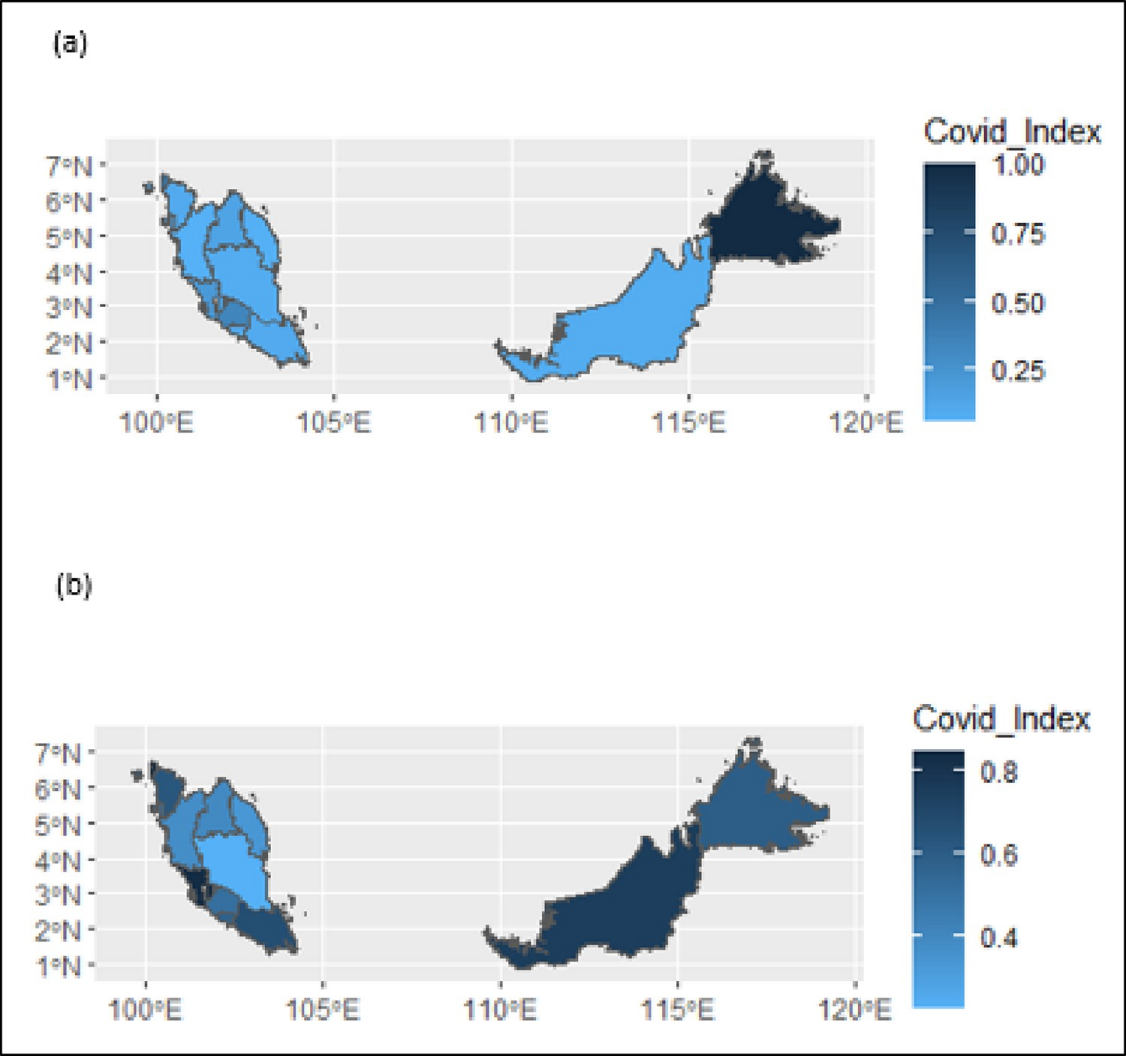
Covid Index for states in Malaysia (a) 2020 and (b) 2021. Note: Dark blue indicates locations with indicators close to one (inadequate situation), whereas light blue indicates regions with indicators close to zero (adequate situation).

Fig 1 depicts the comparative performance of the states in Malaysia throughout the two years investigated in this study. Fig 1(A) depicts the distribution of the efficiency scores across Malaysia in 2020, while Fig 1(B) depicts the distribution of the efficiency scores across Malaysia in 2021. The areas in dark blue indicate the locations with indicators close to one (inadequate situation), whereas those in light blue indicate the regions with indicators close to zero (adequate situation). Table 4 shows the ranks of the states in terms of their performance in 2020 and 2021. According to Table 4, four states, namely, the Federal Territory of Kuala Lumpur, Federal Territory of Putrajaya, Federal Territory of Labuan, and Sabah had the worst efficiency scores in 2020 (SCI: 1.0). Meanwhile, in 2021, the same states, except Sabah, remained with the worst performance during 2021 (SCI: 1.0). Nevertheless, during both years of 2020 and 2021, the four states (Sabah, Federal Territories of Putrajaya, Kuala Lumpur and Labuan) consistently recorded COVID Index scores of more than 0.5, which was considered unsatisfactory. It was observed that these states were clustered in Central (Putrajaya and Kuala Lumpur) and East Malaysia (Sabah and Labuan).

**Table 4:** COVID index in 2020 and 2021.

2020			2021		
States	Rank	COVID Index	States	Rank	COVID Index
**Perak**	1	0.061	Pahang	1	0.221
**Terengganu**	2	0.069	Terengganu	2	0.332
**Pulau Pinang**	3	0.085	Perak	3	0.372
**Pahang**	4	0.088	Kelantan	4	0.389
**Sarawak**	5	0.088	Pulau Pinang	5	0.489
**Kedah**	6	0.104	Negeri Sembilan	6	0.508
**Johor**	7	0.105	Melaka	7	0.572
**Melaka**	8	0.116	Sabah	8	0.593
**Kelantan**	9	0.145	Kedah	9	0.626
**Selangor**	10	0.209	Johor	10	0.667
**Negeri Sembilan**	11	0.341	Sarawak	11	0.750
**Perlis**	12	0.430	Selangor	12	0.843
**Kuala Lumpur**	13	1.000	Perlis	13	0.850
**W.P. Putrajaya**	14	1.000	Kuala Lumpur	14	1.000
**Sabah**	15	1.000	W.P. Putrajaya	15	1.000
**W.P. Labuan**	16	1.000	W.P. Labuan	16	1.000

Note: The closer the value to 1, the worse the state’s performance in utilizing healthcare resources to combat COVID-19.

These results also showed that the central region was the most affected since all three states in the region (Selangor, Federal Territory of Kuala Lumpur, Federal Territory of Putrajaya) showed an inadequate situation (SCI: >0.75) due to the COVID-19 outbreak (Table 4). This result could be attributed to the inadequate hospital infrastructure in these states, where the healthcare system struggled due to a lack of resources and qualified manpower. In contrast, the state of Pahang maintained a score of less than 0.25 for both 2020 and 2021. The greater disparity indicated a decline in the state’s performance over time, where nearly all the states in Malaysia experienced an increase in their COVID Index scores. The situation in 2021 deteriorated compared to 2020, with only the state of Pahang exhibiting an adequate situation (SCI: <0.24). This was significantly different in comparison to the year 2020, when ten states (62.5%) exhibited an adequate situation (SCI: <0.24).

The difference between the efficiency scores in 2020 and 2021, using the average for the state, is displayed in Table 5. Sarawak had the largest rise of 0.663 in its COVID Index from 2020 to 2021. Sabah was the only state with efficiency scores that improved over time. However, the improvement was insufficient because it only involved a change from the worst situation (SCI: 1.0) into the categories that required attention (SCI: 0.59).

**Table 5:** COVID index comparison between 2020 and 2021.

States	Difference*	Rank	Signal
Sabah	0.407	1	-
W.P. Kuala Lumpur	0.000	2	0
W.P. Putrajaya	0.000	3	0
W.P. Labuan	0.000	4	0
Pahang	-0.134	5	+
Negeri Sembilan	-0.167	6	+
Kelantan	-0.244	7	+
Terengganu	-0.263	8	+
Perak	-0.311	9	+
Pulau Pinang	-0.404	10	+
Perlis	-0.419	11	+
Melaka	-0.456	12	+
Kedah	-0.522	13	+
Johor	-0.562	14	+
Selangor	-0.634	15	+
Sarawak	-0.663	16	+

Note: The value acquired from the difference of SCI value from 2020 and 2021. The symbol (-) indicates improved efficiency while (+) indicates deteriorating efficiency.

## Discussion

During the COVID-19 outbreak in Malaysia, healthcare resources in all states were analysed using the COVID Index. This study attempted to assess the effectiveness of the states and Federal Territories in Malaysia during the COVID-19 crisis in 2020 and 2021. This study also found that the Central Region was the most affected since all three states in the region (Selangor, Federal Territory of Kuala Lumpur, Federal Territory of Putrajaya) showed an inadequate situation (SCI>0.75) due to the COVID-19 outbreak. This can be explained by the high population density in this region [18]. The COVID Index suggests that policymakers should pay more attention to states in the Central Region (Selangor, Federal Territory of Kuala Lumpur, Federal Territory of Putrajaya). A comparison of the COVID Index indicated that the situation in Malaysia deteriorated between 2020 and 2021. Pahang was the most effective state in the management of the COVID-19 outbreak, whereas the Central Region was the most affected, since all three states in the region (Selangor, Federal Territory of Kuala Lumpur, Federal Territory of Putrajaya) showed an inadequate situation (SCI: >0.75) due to the COVID-19 outbreak. The lack of an active crisis committee, a system for organizing medical personnel, training courses, new technologies, human resources, and a shortage of medical equipment were among the most serious issues faced in responding to this outbreak. The same scenario was observed in Brazil, as reported by Ferraz et al. [9].

Four main findings should be looked into. First, the state that improved its performance from 2020 and 2021 (Sabah); second, the state with a deterioration in its performance (Sarawak); third, the states that maintained an adequate situation (Kuala Lumpur, Putrajaya and Selangor); and lastly, the state that maintained its best performance in both years (Pahang). It is worth noting that these four observations had to be analysed closely to get a better understanding. For the first observation, the improvement in the performance of the state of Sabah might have been due to the increased attention by the Ministry of Health, since during 2020, Sabah experienced one of the most serious outbreaks. This outbreak was believed to have been caused by the Sabah state elections [19]. After the outbreak was identified, the government sent its resources to Sabah and implemented serious interventions to control the outbreak. The interventions showed positive results when Sabah improved its situation in 2021. For the second observation, there are several possible explanations as to why the performance of Sarawak deteriorated in 2021 compared to in 2020. The increased COVID Index might have been because the interventions that were conducted in this state were less effective. Sarawak is the fourth most populous state in Malaysia after Selangor, Johor and Sabah [20]. However, the available healthcare resources (isolation and treatment beds, physicians and nurses) did not meet the optimum requirements. In other words, these resources did not follow the appropriate ratio. Low level of resources to population ratio poses a threat in causing the failure of outbreak control in a location. These locations will become burdened during a sudden increase in cases. These results are consistent with those reported by Su et al. [21], who found that abrupt rises in cases led to a decline in the effectiveness of outbreak interventions in the study area. For the third observation, Selangor, Kuala Lumpur and Putrajaya remained the most burdened states in terms of their COVID Index probably because of their high population. Selangor is the most populated state in Malaysia, followed closely by Kuala Lumpur and Putrajaya. Locations with large populations will be the most burdened in terms of outbreak control. Even after resources have been increased in these locations, the outbreak control measures might still be unsuccessful due to the overwhelming number of cases. This is due to the resources to cases ratio being still low and therefore unable to efficiently control the outbreaks. Another possible explanation is that the high population density makes it harder for physical distancing to be complied with and therefore causing an increase in cases. A similar pattern of results was obtained by Lupu & Tiganasu [5] in which states with more efficient medical systems has low overall efficiency in controlling the outbreaks.

A task force, known as the ’Greater Klang Valley Special Task Force’, was formed to control the outbreak in these states [22]. The Greater Klang Valley Special Task Force (GKVSTF) was established to alleviate the strain that the rising number of COVID-19 cases was imposing on the healthcare infrastructure of the region [23]. This strategy was successful in increasing not only the bed capacity, but also the oxygen supply, related equipment, and staff resources. In addition, this strategy was also aimed at thinning down the crowds at Covid-19 Assessment Centres (CAC) by implementing virtual CACs to reduce manpower demands as well as to maintain physical distancing.

The fourth important observation was that Pahang maintained its best performance for both years of 2020 and 2021 due to its good resources and manpower-to-population ratio. This could also have been due to its low population density as well as the effect of the implementation of the Movement Control Order [24]. The Movement Control Order involved a travel restriction strategy, which succeeded in reducing the healthcare burden in managing the COVID-19 outbreak, as reported in several studies [25–27].

By using the COVID Index, governments will be able to determine whether locations require new investments, the hiring of health professionals, and the reallocation of resources. In addition, this index will assist hospital administrators to identify potential underutilized resources, which is critical for resource reallocation. It is suggested that the COVID Index is a useful tool for countries with imbalanced regions, particularly developing nations, and that it might be used long after the epidemic has passed. This measure can aid policymakers and hospital administrators in regional and national hospitals in planning to optimize patient care. In addition, proper crisis management requires well-planned crisis management, internal and external crisis coordination in the organization, especially with the use of new technologies, identification of capabilities to respond to crises through proper reinforcing and organizing of human resources, and provision of the required training.

The timeframe for the data collection limited the applicability of the research results. Increasing or lowering the number of units in the system was also one of the limitations of this research, as changing the research community might influence the results. The limitation of this study was the absence of more effective performance evaluation markers. This method is merely a mathematical technique based on linear programming and is incapable of analysing the qualitative properties of decision units. This work differed from past research in terms of: First, the COVID-19 cases and mortality were compared to the healthcare resources required for handling this disease; Second, a comparison of the COVID Index was used to evaluate the effectiveness of a state in handling the COVID-19 outbreak over the course of two years; Lastly, there have been no studies so far that examined the relative effectiveness of healthcare resources in managing the COVID-19 epidemic in Malaysia over a period of two years. For this reason, the primary objectives of this study were to bridge this gap in the existing studies. The COVID-19 epidemic spawned a scenario that posed a challenge to healthcare resources in Malaysia. Policymakers, public authorities, and hospital administrators were forced to contend with a lack of these resources. To offer treatment to affected individuals, reduce deaths, and prevent the collapse of the health network as a result of a COVID-19 outbreak, it is essential to examine healthcare resources and ensure their sufficiency. This risk scenario for health systems necessitates tools to monitor and forecast a possible collapse in regional health networks, which would allow competent authorities to mitigate the burden on health systems based on the past experiences of countries that recently emerged from the peak of the contagion curve. Such measures would include the construction of field hospitals, the fortification of lockdowns, along with financial measures to support the health system and protect businesses, coordination between the national and regional governments to ensure new powers over health services, transportation, and internal affairs, and the granting of the authority for law enforcement to the military [28]. In addition, the COVID Index is suitable for reviewing hospital structures during a second COVID-19 wave, other outbreak scenarios, or even after the outbreak, as the indicator identifies which locations should receive new investments and how to reallocate underutilized health resources.

## Conclusions

In conclusion, the findings of this study point to deterioration in the performance of states over the course of time. Between the years 2020 and 2021, nearly all states in Malaysia had an increase in their COVID Index, which indicated an inadequate situation in managing the COVID-19 crisis. The ranking of the states in Malaysia according to their vulnerability to an outbreak of the COVID-19 is vitally significant for the purposes of assisting the government and policymakers in planning their responses to the outbreak and ensuring that resources are distributed appropriately.
